# Assessment of a cancer genomic profile test for patients with metastatic breast cancer

**DOI:** 10.1038/s41598-022-08925-3

**Published:** 2022-03-21

**Authors:** Ippei Fukada, Seiichi Mori, Naomi Hayashi, Mari Hosonaga, Masumi Yamazaki, Xiaofei Wang, Saori Kawai, Lina Inagaki, Yukinori Ozaki, Kokoro Kobayashi, Fumikata Hara, Takayuki Kobayashi, Arisa Ueki, Tomo Osako, Akiko Tonooka, Kengo Takeuchi, Takayuki Ueno, Toshimi Takano, Shinji Ohno, Shunji Takahashi

**Affiliations:** 1 Genomic Medicine, The Cancer Institute Hospital of Japanese Foundation for Cancer Research, 3-8-31, Ariake, Koto-ku, Tokyo, 135-8550 Japan; 2 Breast Medical Oncology, The Cancer Institute Hospital of Japanese Foundation for Cancer Research, Tokyo, Japan; 3 Division of Cancer Genomics, Japanese Foundation for Cancer Research, Cancer Institute, Tokyo, Japan; 4 Medical Oncology, The Cancer Institute Hospital of Japanese Foundation for Cancer Research, Tokyo, Japan; 5 The Center for Advanced Medical Development, The Cancer Institute Hospital of Japanese Foundation for Cancer Research, Tokyo, Japan; 6 Clinical Genetic Oncology, Cancer Institute Hospital, Japanese Foundation for Cancer Research, Tokyo, Japan; 7 Division of Pathology, Cancer Institute, Japanese Foundation for Cancer Research, Tokyo, Japan; 8 Department of Pathology, Cancer Institute Hospital, Japanese Foundation for Cancer Research, Tokyo, Japan; 9 Pathology Project for Molecular Targets, Cancer Institute, Japanese Foundation for Cancer Research, Tokyo, Japan; 10 Breast Surgery, The Cancer Institute Hospital of Japanese Foundation for Cancer Research, Tokyo, Japan; 11 Breast Oncology Center, The Cancer Institute Hospital of Japanese Foundation for Cancer Research, Tokyo, Japan

**Keywords:** Cancer, Breast cancer, Cancer genomics

## Abstract

Comprehensive cancer genomic profile (CGP) tests are being implemented under Japanese universal health insurance system. However, the clinical usefulness of CGP test for breast cancer patients has not been evaluated. Of the 310 patients who underwent CGP testing at our institution between November 2019 and April 2021, 35 patients with metastatic breast cancer whose treatment strategy was discussed by our molecular tumor board within the study period were investigated after exclusion of 2 cases that could not be analyzed. The turn-around time, drug accessibility, and germline identification detection were evaluated. The subtype was luminal in 20 patients (57.1%), triple-negative in 12 patients (34.3%), and luminal-HER2 in 3 patients (8.6%). Actionable gene mutations were detected in 30 patients (85.7%), and 7 patients (20.0%) were recommended for clinical trial participation, with the drug administered to 2 patients (5.7%). Three patients (8.6%) died due to disease progression before the test results were disclosed. We report the results of an initial assessment of the utility of CGP testing for patients with metastatic breast cancer under Japanese universal health insurance system. Conducting CGP tests at a more appropriate time could provide patients with greater benefit from treatments based on their specific gene mutations.

## Introduction

It is estimated that approximately one million people develop cancer each year, and nearly one in every two people develop cancer during their lifetime^1^. Cancer genomic medicine, which enables more effective and efficient diagnosis, treatment, and prevention, is receiving increasing interest. In the United States, then-President Barack Obama announced the "Precision Medicine Initiative" in his State of the Union address in January 2015^2^, and in January 2016, the Vice President led the launch of the "Cancer Moonshot" project, which aimed to develop new diagnostic and treatment methods to overcome cancer^3^. In the UK, Genomics England, established by the UK Department of Health in 2013, is implementing the "100,000 Genomes Project" to analyze the whole genome of patients with cancer and rare diseases. In December 2018, Genomics England announced the completion of the 100,000 Genomes Project analysis^4^. In addition, in October of the same year, the UK Minister of Health announced the goal of conducting one million whole-genome analyses over the next 5 years.

In Japan, two types of "cancer genomic profile (CGP) tests" (OncoGuide NCC Oncopanel System and FoundationOne CDx Cancer Genome Profile) entered full-scale application under the universal health insurance system in June 2019^5–8^. In accordance with the Clinical Practice Guidance for Next-Generation Sequencing in Cancer Diagnosis and Treatment (edition 2.1), the gene panel tests covered by insurance include those for patients with solid tumors for whom no standard treatment is available or for whom standard treatment has been completed (including those who are expected to complete such treatment)^8–10^. In addition, for the purpose of consolidating precision oncology treatment with quality control and assurance in designated hospitals, the Ministry of Health, Labour and Welfare (MHLW) originally designated 12 cancer genomic medicine core hospitals, 33 hub hospitals, and 180 cooperative hospitals in April 2021^8,11^.

CGP tests use next-generation sequencing and other technologies to analyze a large number of cancer-related genes simultaneously, which is expected to assist in determining treatment strategies, thereby improving cancer treatment outcomes. In the SAFIR01 study, 423 patients with metastatic breast cancer underwent gene panel testing, which identified actionable mutations in 195 patients (46%). The mutations commonly identified included *PIK3CA* mutation (25%), *CCND1* amplification (18%), and *FGFR1* amplification (12%), followed by *AKT1* mutation, *EGFR* amplification, and *MDM2* amplification. A total of 55 of these patients (13%) were treated based on identified gene mutations^12^.

The TOP-GEAR (Trial of Onco-Panel for Gene-profiling to Estimate both Adverse events and Response) project, a study using CGP testing, reported patient access to therapeutic agents after standard treatment in Japan. From May 2016 to May 2017, approximately half of the patients who participated in the project and were able to undergo genetic analysis were found to have abnormalities in actionable genes, and 25 patients (13.4%) received therapeutic drugs tailored to their individual gene mutations, of which only one involved breast cancer^13^.

The CGP tests are being implemented in Japan under the universal health insurance program, but many issues remain to be resolved, such as the limited number of implementation targets after standard treatment, the complexity of the national insurance requirements, management of the molecular tumor board (expert panel), and drug accessibility. Therefore, the clinical usefulness of CGP testing for breast cancer patients has not been evaluated. The purpose of the present study was to evaluate these issues at our institute under Japanese universal health insurance system.

## Patients and methods

### Patients

Of the 310 patients who underwent CGP testing at our institution between November 2019 and April 2021, those with metastatic breast cancer whose treatment plan was discussed by the molecular tumor board within the study period were retrospectively studied, except for patients whose analysis failed. In Japan, insurance coverage for CGP tests is restricted to patients with advanced solid tumors exhibiting disease progression during standard therapy or patients for whom there are no appropriate standard treatments, including patients with rare cancers and carcinomas of unknown primary origin.

The study was approved by the Institutional Review Board of our institution (2021-1195), and data were collected in compliance with the ethical requirements of our institution.

### Pathological assessment

Immunohistochemical subtypes were determined based on the primary tumor. Tumor sections were stained with hematoxylin–eosin (HE) and immunohistochemically examined for estrogen receptor (ER), progesterone receptor (PgR), and human epidermal growth factor receptor 2 (HER2) expression. Immunohistochemical assessment of ER and PgR expression was performed using antibodies against ER, clone 1D5 (Dako Japan Inc., Tokyo, Japan), and for PgR, clone PgR636 (Dako Japan Inc.). Positive reactions for ER and PgR were defined as nuclear staining in ≥ 1% of cancer cells, and negative reactions were defined as staining in < 1% of cancer cells. Hormone receptor positivity was defined as positivity in ER and/or PgR staining. HER2 positivity was defined as HER2 protein 3+ or HER2 gene amplification, according to the ASCO/CAP guidelines^14^.

After combining ER, PgR, and HER2 data, patients were classified into four subtypes, defined as follows: luminal subtype, ER+ and/or PgR+, HER2−; luminal HER2 subtype, ER+ and/or PgR+, HER2+; HER2 subtype, ER−, PgR−, HER2+; and triple-negative subtype, ER−, PgR−, HER2−.

### CGP test

The FoundationOne CDx Cancer Genomic Profile was used in this study^5^. The FoundationOne CDx Cancer Genomic Profile (Foundation Medicine) is a CGP platform that applies next-generation sequencing to in vitro diagnostics with a hybrid capture-based target enrichment approach and whole-genome shotgun library construction in order to identify gene substitutions, insertions and deletions (indels), copy number alterations, and select rearrangements. The FoundationOne CDx Cancer Genomic Profile detects alterations in a total of 324 genes, including all coding exons of 309 cancer-related genes (substitutions, insertions and deletions, copy number alterations) and select intronic regions of 36 commonly rearranged genes. In addition, the FoundationOne CDx Cancer Genomic Profile simultaneously profiles for tumor mutation burden (TMB) as well as microsatellite instability (MSI) status.

The rates of CGP testing success, turn-around time (TAT), drug accessibility, and secondary finding detection were evaluated.

The pathologist selected the representative block from the patient's stored formalin-fixed paraffin embedded (FFPE) specimens and estimated the tumor content in the HE specimen. In addition, 10 to 25 slices of 5 μm were prepared to achieve a tumor volume of at least 25 mm^2^, which is the volume required for analysis, and submitted for CGP test.

### Molecular tumor board (expert panel)

The molecular tumor board of our institute discussed the reports from CGP tests (FoundationOne CDx) in conjunction with the Center for Cancer Genomics and Advanced Therapeutics and generated final reports for the patients. The molecular tumor board consists of physicians specializing in cancer pharmacotherapy; physicians with expertise in genetic medicine; genetic counselors; pathologists; physicians with expertise in molecular genetics and cancer genomic medicine; and those with expertise in bioinformatics. The evidence levels for therapeutic efficacy were categorized as A to F, following the JSMO/JSCO/JCA guideline^10^. The actionable gene mutations were defined as following, predictive evidence level D and more^10^ and high-tumor mutation burden (after approval of pembrolizumab by FDA).

### Ethics approval and consent to participate

This retrospective study was approved by the Institutional Review Board of the Cancer Institute Hospital of the Japanese Foundation for Cancer Research (2021-1195). Comprehensive written informed consent for the use of specimen materials was obtained from all patients participating as subjects in this study. In addition, we also used the official website of Cancer Institute Hospital as an opt-out method because of requirement from Japanese law in a non-invasive observational trial such as the present study. And all procedures performed in studies involving human participants were in accordance with the Declaration of 1964 Helsinki and the Ethical Guidelines for Medical and Health Research involving Human Subjects by Ministry of Health, Labour and Welfare (MHLW), the Ministry of Education, Culture, Sports, Science and Technology (MEXT), and the Ministry of Economy, Trade and Industry (METI) of Japan.

## Results

### Patient characteristics

**Figure 1:**
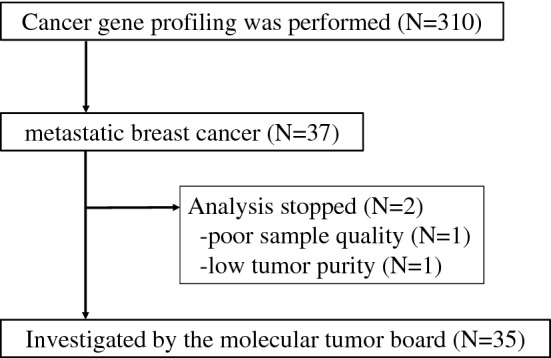
Study consort diagram.

The CONSORT diagram of the study is shown in Fig. 1. Characteristics of the 35 patients investigated in detail are listed in Table 1. All evaluated patients had invasive breast cancer. The median age of the patients was 56 years (range 32–81 years). Evaluation of histological type as established by needle biopsy or surgical specimen showed that more than half of the patients had invasive ductal carcinoma (54.3%). There were 7 specific types, including 4 cases of invasive lobular carcinoma (11.4%) and one case (2.9%) each of spindle cell carcinoma, mucinous carcinoma, and metaplastic carcinoma. The subtype was luminal in 20 patients (57.1%), triple-negative in 12 patients (34.3%), and luminal-HER2 in 3 patients (8.6%).

**Table 1:** Patient characteristics.

	n = 35	
	No	%
**Age (y)**		
Median	56	
Range	32–81	
50 >	10	28.6
50 and more	25	71.4
**Histological type**		
Invasive ductal carcinoma	26	74.3
Invasive lobular carcinoma	4	11.4
Metaplastic carcinoma	2	5.7
Mucinous carcinoma	1	2.9
Apocrine carcinoma	1	2.9
Non-invasive ductal carcinoma	1	2.9
**Subtype**		
Luminal	20	57.1
Luminal-HER2	3	8.6
Triple Negative	12	34.3
**The number of pretreatment regimens**		
1	5	14.3
2	4	11.4
3	1	2.9
4	7	20.0
5	4	11.4
6	3	8.6
7	3	8.6
8	2	5.7
9	2	5.7
10 and more	4	11.4
**The specimens submitted for gene panel testing**		
Primary breast sites	23	65.7
Biopsies from metastatic sites	12	34.3
**Metastatic sites**		
Axillary lymph nodes	2	16.7
Chest wall	2	16.7
Lung	2	16.7
Skin	1	8.3
Liver	1	8.3
Pleural fluid cell block	1	8.3
Ovary	1	8.3
Uterus	1	8.3
Bone	1	8.3

The median number of pretreatment regimens, including regimens currently being treated, was 5 (range 1–16). In 5 cases, CGP testing was performed during the initial treatment of metastatic breast cancer, and 4 patients (11.4%) had ≥ 10 pretreatment regimens.

The specimens submitted for CGP testing included 23 primary breast sites (65.7%) and 12 biopsies from metastatic sites (34.3%). The biopsy specimens included one case of pleural fluid cell block.

### Type and frequency of identified genetic variants

**Figure 2:**
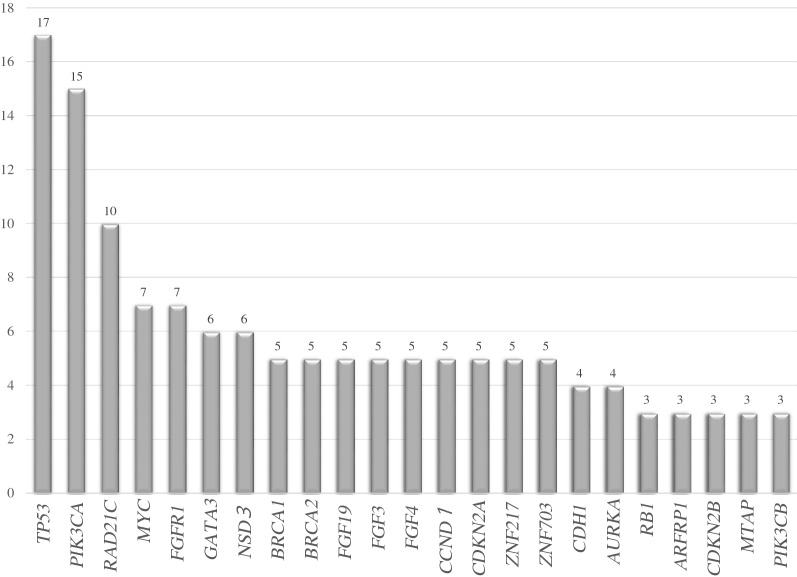
Type and frequency of identified genetic variants. The gene mutations and amplifications detected in this study are shown.

The gene mutations and amplifications detected in this study are shown in Fig. 2. As reported previously, *TP53* and *PIK3CA* mutations were found in more than 40% of the cases. *BRCA1/2* and *RAD21C* mutations were also found in approximately 30% of cases in this study.

**Figure 3:**
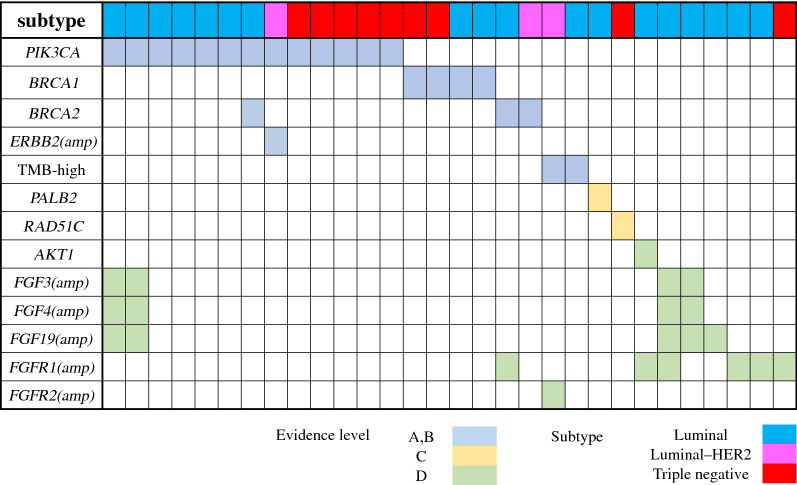
Actionable gene mutations identified in each case. Patients with actionable gene mutations are listed (n = 30).

Thirty cases (87.5%) involved actionable gene mutations, as shown in Fig. 3. These included 21 cases (60.0%) of gene mutations, 11 cases (31.4%) of gene amplification, and 2 cases (5.7%) involving tumor mutation burden (TMB) of ≥ 10 Mb; however, no cases involved gene fusion.

### Turn-around time

The median turn-around time from obtaining consent to the molecular tumor board was 44 days (range 29–93 days), and from obtaining consent to explaining the CGP test results was 56 days (range 42–105 days).

### Drug accessibility rate

**Figure 4:**
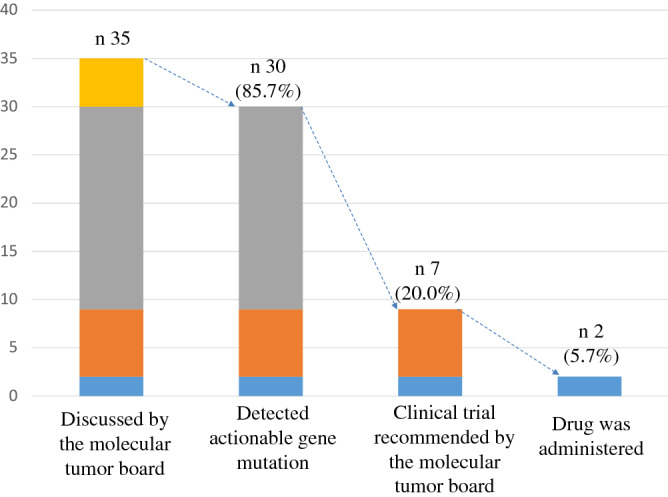
Drug accessibility rate. Drug accessibility status is shown. Of the 30 patients in whom actionable gene mutations were detected, only 2 patients (5.7%) received genome-matched treatment.

Among the 35 patients investigated in detail, the molecular tumor board recommended treatment according to the gene mutation for 7 patients (20%), but only 2 patients (5.7%) participated in clinical trials, as 5 patients were unable to undergo treatment for their mutations due to disease progression (Fig. 4). The 7 cases in which the molecular tumor board recommended treatment according to the gene mutation are shown in Table 2.

**Table 2:** Molecular tumor board treatment recommendations.

Case	Age	Subtype	No. of previous treatments	Gene	Candidate for therapy	Status of treatment
1	58	Luminal	11	*FGFR1* (amp)	TAS120	Participation in clinical trial
2	68	Luminal	9	*FGFR1* (amp)	TAS120	
3	39	Triple negative	1	*RAD51C*	Olaparib	Unable to participate in the recommended clinical trials due to diseases progression
4	50	Luminal	5	*FGF3/4/19* (amp)	TAS120	
5	45	Triple negative	2	*TP53*	AMG650	
6	37	Triple negative	2	*TP53*	AMG650	
				*FGFR1*(amp)	TAS120	
7	62	Luminal-HER2	10	*FGFR2*(amp)	Erdafitinib TAS120	Pre-treatment ongoing
				TMB-high	Nivolumab Pembrolizumab	

With regard to the status of CGP test result disclosure, 3 patients (8.6%) died due to disease progression before the test results were disclosed; thus, the results of the CGP tests could not be explained.

### Germline findings

There were 9 cases in which germline pathological variants were suspected, among which 6 cases had known pathologic variants of *BRCA1/2* before the CGP test (Table 3). In the CGP test, *RAD51C*, *BRCA1*, and *PALB2* were identified as newly suspected germline variants in one case each. In a patient with triple-negative breast cancer suspected of having germline variants in *RAD51C*, confirmatory testing by specific site analysis revealed pathological variants in *RAD51C*. Patients with suspected germline variants in *BRCA1* were confirmed negative by BRAC Analysis.

**Table 3:** Cases involving suspected germline pathological variants.

Case	Age	Subtype	Gene	Genetic counseling	Confirmatory test	Note
1	39	Triple negative	*RAD51C*	Yes	Yes	Specific site analysis revealed pathogenic variation
2	37	Triple negative	*BRCA1*	Done	Known	
3	57	Luminal	*BRCA2*	Done	Known	
4	32	Luminal	*BRCA2*	Done	Known	
5	39	Luminal	*BRCA1*	Done	Known	
6	65	Triple negative	*BRCA1*	Yes	Yes	Negative by BRCA analysis
7	38	Luminal	*BRCA1*	Done	Known	
8	42	Luminal-HER2	*BRCA2*	Done	Known	
9	52	Luminal	*PALB2*	None	None	Confirmatory tests could not be performed due to worsening of the patient's condition

## Discussion

We report here for the first time the initial assessment of the utility of CGP testing for patients with metastatic breast cancer under Japanese universal health insurance system. In the first 18 months after implementation of reimbursement for CGP tests, 310 CGP tests were conducted in our institute during the study period and thirty-five patients with metastatic breast cancer were evaluated. Actionable gene mutations were detected in 30 patients (85.7%), and participation in a clinical trial was recommended to 7 patients (20.0%). The drug was administered to only 2 patients (5.7%), as 3 patients (8.6%) had newly suspected germline pathological variants, and the results were subject to disclosure.

There have been many reports on treatment strategies for metastatic breast cancer using CGP testing. Huang et al. reported that genomic landscape of 312 patients with breast cancer using the FDA-approved Foundation One CDx assay, and the top 5 detected genes in descending order were *PIK3CA*, *TP53*, *RAD21*, *NBN*, and *CCND1* in HR+/HER2− disease subset, *TP53*, *CDK12*, *PIK3CA*, *MYC*, and *RAD21* in HER2+ disease subset, *TP53*, *RAD21*, *MYC*, *PIK3CA*, and *NBN* in TNBC disease subset^15^. Wheler et al. reported that of the 339 patients who successfully performed analysis of CGP testing, 122 patients (36.0%) received treatment according to the gene mutations^16^. Although the results of the genomic landscape of breast cancer using Foundation One CDx assay was almost the same as those of our study, there was a large difference in the drug accessibility rate.

In Japan, several studies using CGP test have been reported. Firstly, as an analysis for research purpose, Kawaji et al. reported the results of Foundation One CDx assay from 109 Japanese breast cancer patients and also reported 54% of patients had clinical evidence level A mutations according to the consensus of three major Japanese cancer related societies and the Center for Cancer Genomics and Advanced Therapeutics^17^. However, the report by Kawaji et al. was for research purposes and did not evaluate the achievement rate of treatment according to gene mutations or the usefulness of CGP testing in actual clinical practice. Secondly, as for the current status of CGP testing under universal health insurance in Japan, 805 patients underwent CGP testing between June and October 2019, and it was reported that 10.9% of these patients were then treated based on their gene mutations^18^. Furthermore, according to a report from 11 core hospitals for cancer genomic medicine, among the 747 cases in which CGP tests were conducted (from June 2019 to January 2020), 28 patients (3.7%) received genome-matched treatment^19^. Of these 28 patients treated according to their gene mutations, only 2 patients had breast cancer, and both were treated with an mTOR inhibitor for a *PIK3CA* mutation in that study^19^. However, since mTOR inhibitor are not approved for PIK3CA mutations, it is not appropriate to evaluate these cases as having been treated strictly according to the gene mutations.

Although there are few previous reports on turn-around time, we reported the median turn-around time from obtaining consent to the molecular tumor board was 44 days, and from obtaining consent to explaining the CGP test results was 56 days. The Foundation Medicine estimates that it takes about 12 days from receipt of specimens to return of analysis results to medical institutions. The reasons for the longer TAT might be as follows. The first reason is that it took several days to a week to transport specimens from Japan to Foundation Medicine. The second reason is that the results of CGP testing are required to be reviewed by molecular tumor board before being returned to the patient under national health insurance in Japan. The third reason is that medical fees (480,00 yen) can only be charged when the results of CGP testing are explained in an outpatient visit. In our study, there was a patient who required 92 days to explain the results in the outpatient clinic after discharge due to prolonged inpatient treatment due to disease progression.

The novelty and importance of our report in comparison with these previous foreign and Japanese reports are as follows. Firstly, our report not only showed low drug accessibility rate for metastatic breast cancer with CGP testing under Japanese universal health insurance system, but also discusses in detail the reasons for the cases in which treatments according to the gene mutations were recommended by molecular tumor board but could not be administered. Secondly, we examined in detail the turn-around time of CGP testing performed under Japanese universal health insurance system. Thirdly, we revealed that approximately 8% of patients died due to disease progression before the test results were disclosed; thus, the results of the CGP tests could not be explained.

Possible causes of these issues include the indication and official pricing system of reimbursement of CGP testing covered by Japanese universal health insurance system. The indication for CGP testing covered by insurance in Japan is restricted to patients with advanced solid tumors exhibiting disease progression during standard therapy (including those expected to be completed) or patients for whom there are no appropriate standard treatments, including those with rare cancers and carcinoma of unknown primary origin^8–10^. Furthermore, reimbursement for the cost of a CGP test is 560,000 yen, paid in two steps. The first reimbursement is 80,000 yen after applying for the informed consent for CGP testing and preparation of tumor samples. The second reimbursement of 480,000 yen is paid when the patient receives an explanation of the CGP test results in an only outpatient visit after assessment by the molecular tumor board.

As mentioned above, the results of our analysis were almost identical to the genomic landscape of breast cancer reported by Huang RSP et al. Even though CGP testing is now limited to after the completion of standard treatment due to insurance requirements in Japan, experts in each field examined the results of CGP testing in detail in molecular tumor board and could provide new treatment options, such as those available in clinical trials to 7 patients (20.0%). However, 5 (14.3%) of these patients were unable to undergo genome-matched treatment due to disease progression after the CGP test. Therefore, our study revealed that the benefits of CGP testing might not be fully extended to patients with metastatic breast cancer due to the indication for CGP testing covered by insurance in Japan.

Although the definition of the standard treatment for metastatic breast cancer prior to CGP testing remains controversial, the treatments strongly recommended in the guidelines of the Japanese Breast Cancer Society are considered candidates in Japan^20^. However, the patient may be eligible for CGP testing without all standard therapies, depending on the efficacy and safety of previous therapies, the patient’s general condition, and patient preference in consideration of the 6- to 8-week turnaround time from submission of tumor tissue to return of analysis results. Furthermore, the Consensus clinical practice guidance for CGP testing in Japan was updated and now recommends CGP test would be recommended, regardless of the line of treatment^21^. Therefore, our results suggest that CGP testing conducted at the appropriate time and based on the efficacy of previous therapy, the patient’s general condition, and patient preference might improve the drug accessibility rate.

It is also very important to conduct multiple CGP testing as needed to evaluate the acquired mutations and resistance-acquired mutations due to therapeutic modification and utilize them in treatment strategies^22^. Although CGP testing are only allowed to be used once per patient under Japanese universal health insurance system, multiple CGP testing, including the CGP test using blood samples, might provide greater benefits to patients.

As mentioned above, CGP testing is currently restricted to patients who finished standard therapies in order to avoid unnecessary investigations and reduce the burden for the molecular tumor board. However, patients who finished standard therapies for metastatic or recurrent cancer tend to have poor prognosis due to disease progression. Therefore, it is important to perform the CGP test at the diagnosis of metastatic breast cancer. Also, changes to the insurance system should be considered, in addition to increasing the number of clinical trials and promoting efforts to standardize quality while reducing the burden on the molecular tumor board.

In summary, it has been almost 2 years since CGP testing covered by the universal health insurance system was first introduced in Japan, and implementation of cancer genomic medicine is progressing. We reported here the initial assessment of CGP testing for patients with metastatic breast cancer and revealed that the percentage of patients who could reach clinical trials is low. In our sample group, a number of patients died before the test results were disclosed, although more than 80% of the patients had actionable mutations. In order to make CGP testing clinically valuable for patients with metastatic breast cancer, it will be necessary to solve problems associated with the current program of insurance coverage for CGP testing in Japan.

## Conclusion

We reported here the initial assessment of the utility of CGP testing for patients with metastatic breast cancer. In our institute, 5.7% of patients underwent genome-matched treatment, and 8.5% were found to have newly suspected germline variations. Conducting CGP tests at a more appropriate time could provide patients with greater benefit from treatments based on their specific gene mutations.

## Data Availability

The datasets collected during and/or analyzed during the current study are available from the corresponding author on reasonable request.
